# Device and method matter: A critical evaluation of eccentric hamstring muscle strength assessments

**DOI:** 10.1111/sms.13569

**Published:** 2019-10-30

**Authors:** Hans‐Peter Wiesinger, Christoph Gressenbauer, Alexander Kösters, Manuel Scharinger, Erich Müller

**Affiliations:** ^1^ Department of Sport and Exercise Science University of Salzburg Salzburg Austria

**Keywords:** angle of peak torque, bilateral strength ratio, dynamometer, eccentric peak torque, nordic hamstring device, reproducibility, sample‐based calibration validity, work

## Abstract

Equivocal findings exist on isokinetic and Nordic hamstring exercise testing of eccentric hamstring strength capacity. Here, we propose a critical comparison of the mechanical output of hamstring muscles as assessed with either a dynamometer (IKD) or a Nordic hamstring device (NHD). Twenty‐five volunteers (26 ± 3 years) took part in a counterbalanced repeated‐measures protocol on both devices. Eccentric peak torque, work, angle of peak torque, bilateral strength ratios, and electromyography activity of the biceps femoris long head, semitendinosus and gastrocnemius muscles were assessed. There was a very poor correlation in eccentric peak torque between the devices (*r* < 0.58), with a systematic and proportional bias toward lower torque values on the IKD (~28%) and a high typical error (~19%) in IKD and NHD measurements comparison. Furthermore, participants performed a higher total eccentric work on IKD, reached peak torques at greater knee extension angles, and showed a greater side‐to‐side strength difference compared to the Nordic hamstring exercise. Gastrocnemius muscle activity was lower during the Nordic hamstring exercise. Reliability was low for work on NHD and for angle of peak torque and bilateral strength ratios on either device. We conclude that the evaluation of eccentric knee flexor strength depends on the testing conditions and even under standardized procedures, the IKD and NHD measure a different trait. Both tests have limitations in terms of assessing strength differences within an individual, and measurements of the angle of peak torque or side‐to‐side differences in eccentric knee flexor strength revealed low reliability and should be considered with caution.

## 1 INTRODUCTION

High eccentric knee flexor strength is crucial to improve athletic performance^1^ and reduce the vulnerability of hamstring muscles.^2^, ^3^ Consequently, strength tests to determine muscle function and performance are well established, but strains or lesions of the hamstring muscles remain endemic and recur at high rates in elite and recreational athletes.^4^, ^5^ Hence, there is a high demand to validate and optimize training and screening procedures to counteract the economic^6^, ^7^ and health‐related burdens of hamstring injuries.

Stationary isokinetic dynamometers (IKD) are recognized as the gold standard for assessing eccentric knee flexor strength,^8^, ^9^, ^10^ but they lack practical utility compared to the Nordic hamstring device (NHD).^11^ Since researchers have assumed that both devices measure the same trait,^10^ the NHD has been quickly established as a tool to detect and modify strength deficits or side‐to‐side imbalances,^12^, ^13^ to monitor exercise‐related strength progress,^14^ or to predict recovery time after injury.^15^

Both devices seem to provide a reliable measure of eccentric strength,^9^, ^16^ although a recent study indicated a poor within‐subject correlation (*r* = 0.35), with evidence of a systematic bias toward lower strength values with the Nordic hamstring exercises (analysis of Figure 4 in the cited reference).^10^ However, comparing measures obtained with both devices in previous studies remains difficult, since a number of methodological specificities were unaccounted for. For instance, IKD testing and NHD testing are typically performed under different conditions related to joint velocity^17^ and hip position.^18^ A thorough comparison matching biomechanical parameters of these tests is currently required to compare the mechanical output measured in each of them and to give an exhaustive assessment of their concurrent validity.

Hence, the present study was to determine the concurrent validity of the NHD against the IKD, while also extending analysis of reliability under matched conditions. Device inherent modalities (eg, punctum fixum ‐punctum mobile, unilateral vs bilateral testing) were retained, but experiments were performed counterbalanced, with controlled hip position and test mode speed. The IKD testing was—given the excellent standardization—used as a criterion to validate the portable NHD testing.^19^ By matching hip positions, we expected the hamstring muscles to operate in a more comparable force‐length and force‐velocity relationships.^20^ This standardized measurement approach should help us elucidate or rule out mechanism responsible for device‐specific strength assessments and their suitability for screening, prevention, or rehabilitation procedures. We hypothesized similar knee flexor torque values when measured on the NHD compared to the IKD.

## 2 METHODS

### 2.1 Participants

Twenty‐five healthy male student athletes volunteered to participate in the study (age, 25.5 ± 2.6 years; height, 182 ± 7 cm; and body mass, 79.5 ± 9.8 kg: left/right lower limb length 42.2 ± 1.8 cm/42.1 ± 1.9 cm). The exclusion criteria were a history of hamstring specific resistance training, prior hamstring strain injury, or other self‐reported musculoskeletal, cardiovascular, or neuromuscular impairments that impeded maximal muscle contraction. Participants were verbally contacted, and the purposes, benefits, and risks of testing procedures were given prior to obtaining written informed consent. The study was approved by the Local Research Ethics Committee (EK‐GZ: 12/2017) and was conducted in accordance with the Declaration of Helsinki.

### 2.2 Experimental design

The study design consisted of a counterbalanced three‐session repeated‐measures protocol with 72 hours between each session. Tests on the IKD (D&R Ferstl GmbH) were performed separately on both legs, according to a prior block randomization based on leg dominance. Participants familiarized themselves with the testing procedures one week before the first session and were required to refrain from stimulant ingestion (eg, caffeine) and vigorous activity (eg, running, jumping, resistance training) for 6 and 24 hours prior to testing, respectively. A normal diet was maintained during the study period. Sessions were conducted by the same investigators at a similar time of day (±2 hours).

### 2.3 Eccentric hamstring strength

**Figure 1:**
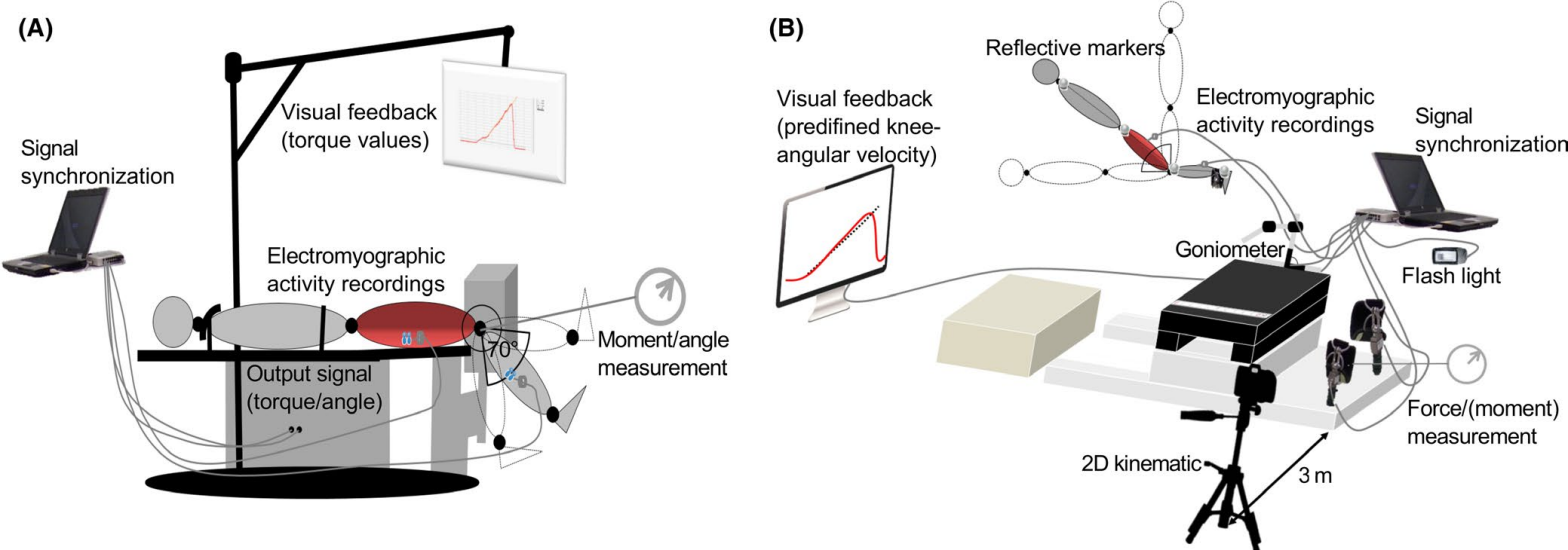
Illustration of the measurement setup in the isokinetic dynamometer (A) and the Nordic hamstring device (B). The IsoMed 2000 dynamometer was calibrated according to the manufacturer's specification, and individual settings determined in the first session were saved by the device‐integrated software. Similarly, the Nordic hamstring device was calibrated using standardized weights and the individual participants’ settings (eg, knee position on the padded board) were recorded in the protocol. The custom‐made Nordic hamstring device, with the load cells secured to a pivot, is undoubtedly comparable to previously used Nordic hamstring devices. The video sequences were synchronized to the Nordic hamstring device parameters with an electrical pulse and a flashlight. Strength, angle, and surface electromyography data were acquired at a 2000 Hz sampling frequency

Figure 1 summarizes the experimental design of this study. Testing sessions were preceded by 10 minutes of supervised cycling at 1.5 W kg^−1^ at a cadence of ~70 rpm on a stationary ergometer (Heinz Kettler GmbH and Co. KG). Two warm‐up trials were done on each diagnostic device, at ~80% of subjectively perceived maximum effort. Exercise modes on the IKD and NHD were standardized with regard to knee angular velocity and hip position. Investigators provided consistent instructions and verbal encouragement throughout each repetition. Participants rested for one minute between contractions. A break of 3 days between tests appeared adequate, as both pilot tests in our laboratory and previous studies^11^, ^21^ have shown that a few maximal (IKD) and supramaximal (NHD) eccentric loads were well tolerated.

Participants were fastened in a supine position and secured to the IKD via adjustable straps and pads across the shoulders, chest, pelvis, and thigh.^22^ Their hip joint angle was set at 0° (0° = full extension), and the knee joint center was carefully aligned with the dynamometer axis of rotation (Figure 1A). Maximum eccentric knee flexor strength was obtained through three afterloaded isokinetic knee extensions. Hence, the dynamometers' lever arm started an −30° s^−1^ fast upward movement (70‐0°, 0° = full knee extension) after exceeding a threshold torque of 20 Nm. This range of motion was selected based on preliminary tests in our laboratory and approximated the mean angle of peak torque achieved on the NHD within this population. Participants were instructed to pull their heel over the entire range of motion as hard and as fast as possible toward the buttocks.

On the NHD, participants were positioned according to previous studies.^9^, ^21^ The midpoints of the ankle braces were positioned above the lateral malleoli, and the load cells (Megatron Elektronik GmbH & Co. KG,) were perpendicular to the participants’ shanks. The rotation axis of the goniometer (Biovision) was aligned with the knee joint, such that the upper stirrups did not touch the thigh in the rectangular starting position. Thus, participants were provided with continuous instantaneous visual feedback of their knee angle. In addition, a video camera (JVC GC‐PX100BEU at 50 Hz) was placed perpendicular to the sagittal plane, capturing reflective markers attached to the participants' lateral malleolus, femoral epicondyle, trochanter major of the femur, and the belly of the deltoideus muscle (Figure 1B). The participants gradually leaned forward from the initial upright position (90° knee flexion) until the gravity‐induced moments exceeded the maximum eccentric knee flexor moment. The arms were crossed across the chest, and the participants were instructed that the hip remained near full extension. Trials were completed at −30° s^−1^ (NHD_30_) and a traditional slowest possible knee angular velocity (NHD_max_).^9^, ^23^ The NHD_30_ trials were repeated if participants were unable to match the −30° s^−1^ forward lean velocity (from visual inspection).

### 2.4 Electromyography

Agonist muscle activation of the biceps femoris long head, semitendinosus and both heads of the gastrocnemius muscles were estimated from surface electromyography recordings of the third session (Electrodes: Ambu^®^ Neuroline 720, 72000‐S/25, Ambu A/S) in accordance with the SENIAM guidelines.^24^ Electromyography data of both legs and NHD_30_ and NHD_max_ were averaged and standardized using two additional bilateral maximal voluntary isometric knee flexions (10°; 0° = full knee extension) and plantarflexions, (anatomical position at 90° ankle joint) for the biceps femoris, semitendinosus, and gastrocnemius.

### 2.5 Data analysis

All data records were synchronized and processed offline using a custom MATLAB code (version R2016a; The MathWorks Inc). The single trial, including either the highest eccentric torque (IKD) or the highest sum of bilateral peak forces (NHD), was saved for further analysis. Nordic hamstring trials in which hip flexion exceeded 20° at any time point and/or NHD_30_ trials with a mean forward lean velocity outside of 20‐40° s^−1^ were discarded. Reflective markers were digitized with a semiautomatic video analysis (Tracker 4.87, physlets.org/tracker/). Knee flexor torque was calculated using the force recorded during NHD trials and the shortest distance between the lateral malleolus and the femoral epicondyle. Knee joint kinetics were offset and smoothed using a digital second‐order, zero‐lag Butterworth filter with a cutoff frequency of 15 Hz. For IKD measures, gravitational and stretch‐induced forces were estimated via measuring the torque during passive rotation of the knee joint (70°‐0°). The angle‐specific passive torque was subtracted from the isokinetic eccentric force‐velocity curves. The eccentric work was calculated as the area under the torque‐angle curves using trapezoidal numerical integration. Side‐to‐side strength differences were obtained after back‐transformation of log‐transformed torque values.^16^ Maximum agonist sEMG amplitude was calculated using the root‐mean‐square of the signal over a 0.5 seconds window around the peak torque. Raw signals were filtered using a second‐order bandpass zero‐lag digital Butterworth filter with cutoff frequencies of 10 and 300 Hz. Additionally, root‐mean‐square values of eccentric contractions were expressed as a percentage of the respective maximal voluntary isometric knee flexion and plantarflexion.

### 2.6 Statistics

Data are expressed as mean ± standard deviation (SD) unless otherwise noted. Bilateral hamstring ratio and data that provided nonuniformity of the residuals were analyzed using a log transformation when appropriate. The mean of log‐transformed data was obtained by antilogging, while the standard deviation was kept as a percent variation or coefficient of variation. If variance‐stabilizing transformation could not be achieved and data provided substantial skewed distribution and/or kurtosis, nonparametric tests were performed. For inter‐device comparisons, hamstring parameters derived during the third session were compared using paired sample *t* tests or Wilcoxon signed‐rank test. Pearson's correlation coefficients were used to determine the relationships between variables related to the IKD and NHD. Magnitudes of correlations were interpreted qualitatively using: *r* < 0.45, impractical; 0.45‐0.70, very poor; 0.70‐0.85, poor; 0.85‐0.95, good; 0.95‐0.995, very good; and >0.995, excellent.^25^ Concurrent validity—with the dynamometer measures as the criterion—was assessed using regression validity analysis.^26^ A proportional bias was examined by analyzing the similarity (or lack thereof) between the linear regression and equality line.^27^ Systematic errors between sessions were detected using a repeated‐measures analysis of variance (ANOVA) with a Huynd‐Feldt correction for sphericity or with a Friedman's ANOVA. Bonferroni or Wilcoxon signed‐rank corrected post hoc tests were performed for significant between‐session effects. Relative and absolute reliability between sessions was assessed using the intraclass correlation coefficient (ICC_3,1_), or the means of the Fisher's *z*‐transformed Spearman correlation, and the typical error of measurement, expressed as a coefficient of variation (CV*_TE_*). An ICC over 0.9 was considered as high, between 0.8 and 0.9 as moderate, and below 0.8 as low.^28^ The minimum detectable change (MDC_95_) was calculated as ± 1.96∙SEM∙$\sqrt{2}$.^29^ Unless otherwise noted, all statistical analyses were performed using IBM SPSS Statistics V.25.0 (SPSS Inc), while figures were generated using the GraphPad Prism 7.03 (GraphPad Software Inc). The level of significance was set at *P* = .05.

## 3 RESULTS

In the NHD tests, participants were unable to resist the body weight‐induced moment until full knee extension (angle of peak torque NHD_max_ ≥ 12.8°, NHD_30_ ≥ 19.8°). Apart from a lower knee angular velocity (12.6 ± 4.3° s^−1^ vs 28.0 ± 4.5° s^−1^; *t* = 15.44, *P* < .001, $\eta_{p}^{2}$ = 0.91) and a higher angle of peak torque of the left leg (*t* = 2.91, *P* = .008, $\eta_{p}^{2}$ = 0.26) in the NHD_max_ than the NHD_30_, no further differences were observed between the NHD test modes (*t* < 1.84, *P* > .078, $\eta_{p}^{2}$ ≤ 0.13). Hence, for the sake of clarity, the intra‐ and inter‐device comparisons were largely limited to the comparison of the IKD and NHD_30_ tests. Yet, all parameters measured by NHD_max_ remain in the table.

### 3.1 Concurrent validity

**Figure 2:**
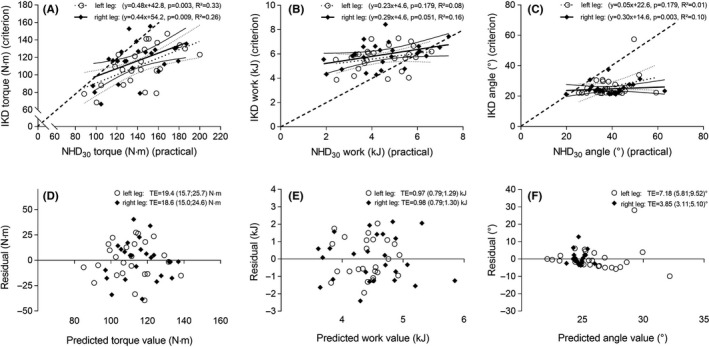
Qualitative assessment of nonlinearity, random error, and systematic error in the relationship between parameters of the dynamometer (IKD) plotted against the Nordic hamstring device (NHD_30_). Dotted and solid lines show the linear regression and their 95% confidence limits, while the dashed line represents the equality line (A, B, C). Plots (D, E, F) show a uniform (homoscedasticity) typical error of the estimate and their 95% confidence limits for the residuals vs predicted. Clarification of the statistical outlier in the angle of peak torque (*z*‐score = 4.22, Figure 2F) revealed that this was a real physiological condition and therefore the data point was not removed. NHD_30_, knee angular velocity of 30° s^−1^

Inter‐device comparison revealed that eccentric peak torque is generally lower when measured on the IKD than the NHD_30_ (left: *t* = 6.87, *P* ≤ .001, $\eta_{p}^{2}$ = 0.66, −21%, right: *t* = 5.03, *P* < .001, $\eta_{p}^{2}$ = 0.51, −16%). However, participants performed a higher total eccentric work (left: *t* = 4.80, *P* < .001, $\eta_{p}^{2}$ = 0.49, +21%, right: *t* = 5.11, *P* < .001, $\eta_{p}^{2}$ = 0.52, +24%) on IKD and reached peak torque at greater knee extension angles (left: *W* = 8.00, *P* < .001, $\eta_{p}^{2}$ = 0.35, +16%, right: *W* = 4.00, *P* < .001, $\eta_{p}^{2}$ = 0.36, +22%). Bilateral strength difference was lower on NHD compared to the IKD (*t* = 2.19, *P* = .039, $\eta_{p}^{2}$ = 0.17),

Eccentric peak torque measures were poorly correlated (left: *r*_(23)_ = 0.58, *P* = .003, right: *r*_(23)_ = 0.51, *P* = .009), and eccentric work (left: *r*_(23)_  = 0.28, *P* = .180, right: *r*_(23)_ = 0.39, *P* = .051) and angle of peak torque (left: *r*_(23)_ = 0.23, *P* = .278, right: *r*_(23)_ = 0.107, *P* = .610) did not correlate. Similarly, a significant linear regression with a proportional bias for either leg (significant differences between the regression line and equality line: left: *t* = 2.56, *P* = .014, right: *t* = 3.20, *P* = .002) was observed for eccentric peak torque in inter‐device testing modes (Figure 2A). The regressions to calibrate eccentric work and angle of peak torque were not significant, with the exception of the angle of peak torque of the right leg (Figure 2B and C).

Hence, when changes in eccentric peak torque measures of the NHD were converted to the IKD measures, the typical error in the estimate was about 19 Nm (Figure 2D). These high typical errors of the estimates of eccentric work and angle of peak torque (Figure 2E and F) may be attributed to the low reliability and the widely nonsignificant linear regressions.

### 3.2 Reliability

Table 1 presents the intra‐device test using the ICC_3,1_ and their 95% confidence limits or mean Spearman correlation, CV*_TE_* and MDC_95_ values on the IKD and NHD for hamstring strength ratios, torque, work, and angle of peak torque. There was no effect for time on the eccentric knee flexor strength for either device. Relative reliability was moderate to high for eccentric peak torque measures (ICC > 0.85), with absolute reliability (CV*_TE_*) ranging from 5.6%‐7.1% and resulting in MDC_95_ between 15.0% and 20.4%. Similarly, there was a moderate reliability of eccentric work for the IKD (ICC > 0.84 and CV*_TE_* < 7.7%), but reliability of eccentric work was poor for the NHD (ICC < 0.74 and CV*_TE_* > 14%). In addition, lower average eccentric work was found in the second trial on the IKD compared to the first (right leg) and third trials (left and right leg), whereas no carryover effects were found using the NHD_30_ testing conditions. Overall, a poor relative reliability was found for the angle of peak torque (ICC < 0.66) and side‐to‐side strength imbalance ratios (ICC < 0.60) assessed by IKD and NHD_30_ tests.

**Table 1:** Reliability of the eccentric peak torque obtained during the isokinetic and Nordic hamstring exercise tests

	Mean ± SD			Main effect *P* value or ᵡ^2^ ($\eta_{p}^{2}$)	ICC_3,1_ or Spearman (95% CI lower; upper)	*CV_TE_*(%)* ± MDC_95_*(%)
	Session 1	Session 2	Session 3			
IKD tests						
Peak torque (N∙m)						
Left leg	108 ± 21	108 ± 22	111 ± 22	.273 (0.05)	0.89 (0.82;0.94)	6.7 ± 18.7
Right leg	115 ± 22	112 ± 21	116 ± 22	.125 (0.08)	0.92 (0.87;0.96)	5.4 ± 15.0
Work (kJ)						
Left leg	5.5 ± 1.0	5.4 ± 1.0	5.7 ± 1.0*	.044 (0.13)	0.84 (0.73;0.91)	7.7 ± 21.4
Right leg	5.9 ± 1.1**	5.6 ± 0.9	5.9 ± 1.0*	.004 (0.22)	0.86 (0.77;0.93)	6.7 ± 18.6
Angle at peak torque (°)						
Left leg^b^	26.4 ± 6.6	25.1 ± 5.3	26.1 ± 7.4	.595 (0.01)	0.54	16.2
Right leg^b^	23.2 ± 3.6**	26.0 ± 5.4	24.7 ± 3.8	.004 (0.10)	0.58	13.8
Bilateral hamstring ratio^a^	0.93 ± 8.6	0.96 ± 13.4	0.95 ± 14.0	.429 (0.04)	0.60 (0.39‐0.76)	8.0 ± 21.4
NHD_max_ tests						
Peak torque (N∙m)						
Left leg	142 ± 30	144 ± 31	146 ± 30	.300 (0.05)	0.90 (0.83;0.95)	6.9 ± 19.1
Right leg	142 ± 29	143 ± 31	148 ± 28	.034 (0.13)	0.94 (0.89;0.97)	5.4 ± 15.0
Work (kJ)						
Left leg	4.2 ± 1.1	4.1 ± 1.3	4.7 ± 1.2**	.004 (0.21)	0.77 (0.62;0.87)	13.6 ± 37.6
Right leg	4.4 ± 1.2	4.4 ± 1.4	4.6 ± 1.4	.351 (0.04)	0.74 (0.58;0.75)	16.0 ± 44.4
Angle at peak torque (°)						
Left leg	36.2 ± 8.6	38.6 ± 8.5	34.5 ± 7.7**	.012 (0.18)	0.75 (0.60;0.86)	11.7 ± 32.5
Right leg	36.4 ± 9.6	38.0 ± 9.4	36.1 ± 8.2	.446 (0.03)	0.67 (0.49;0.81)	14.5 ± 40.3
Bilateral hamstring ratio^a^, ^b^	1.00 ± 10.6	1.01 ± 15.4	0.99 ± 9.9	.432 (0.10)	0.61	9.5
NHD_30_ tests						
Peak torque (N∙m)						
Left leg	146 ± 29	140 ± 29	142 ± 27	.120 (0.09)	0.88 (0.79;0.93)	7.2 ± 19.8
Right leg	143 ± 26	139 ± 24	140 ± 26	.289 (0.05)	0.85 (0.74;0.92)	7.4 ± 20.4
Work (kJ)						
Left leg	4.6 ± 1.0	4.2 ± 1.3	4.4 ± 1.2	.034 (0.14)	0.74 (0.58;0.85)	14.0 ± 38.9
Right leg	4.5 ± 1.3	4.2 ± 1.3	4.5 ± 1.4	.345 (0.04)	0.59 (0.38;0.75)	19.9 ± 55.2
Angle at peak torque (°)						
Left leg	37.3 ± 5.0	41.0 ± 9.5	38.9 ± 8.0	.025 (0.14)	0.66 (0.48;0.81)	11.7 ± 32.6
Right leg	39.1 ± 7.1	41.1 ± 9.5	39.2 ± 8.7	.382 (0.04)	0.49 (0.26;0,69)	15.4 ± 42.8
Bilateral hamstring ratio^a^	1.01 ± 11.2	1.00 ± 10.8	1.02 ± 8.4	.745 (0.01)	0.41 (0.17;0.63)	7.5 ± 20.7

Abbreviations: CV*_TE_*, CI, confidence interval; typical error as a coefficient of variation; ICC, intraclass correlation coefficient; IKD, isokinetic device; MDC, minimal detectable change; angle of 0° corresponds to full knee extension; NHD_30_, Nordic hamstring test at a lean forward velocity of ~30° s^−1^; NHD_max_, Nordic hamstring test at low lean forward velocity.

a For clarity, means of the log‐transformed data were transformed back and standard deviations were kept as a coefficient of variation. Statistical analyses were done with log‐transformed data.

b Data show the Chi‐square and Spearman correlation value.

* *P* < .05.

** *P* < .01.

*** *P* < .001 compared with session 2.

## 4 ELECTROMYOGRAPHY

**Figure 3:**
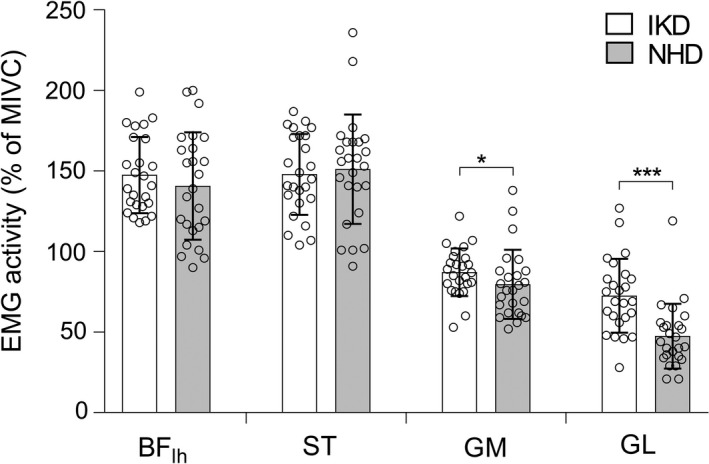
Mean ± SD of the hamstring electromyography (EMG) activity (expressed in percentage of maximal isometric voluntary contraction) on the isokinetic dynamometer (IKD) and Nordic hamstring device (NHD). BF_lh_, biceps femoris long head; ST, semitendinosus; GM, gastrocnemius medialis; and GL, gastrocnemius lateralis. *** *P* < .001 * *P* < .05 between IKD and NHD

There was no difference in the extent to which the biceps femoris and semitendinosus muscles were activated during isokinetic dynamometry and NHD (*P* > .273, $\eta_{p}^{2}$ ≤ 0.05) tests. However, gastrocnemius medialis and gastrocnemius lateralis activation was lower during NHD trials compared to the isokinetic measurement (*t* = 2.45, *P* = .022, $\eta_{p}^{2}$ ≥ .20, −9% and *t* = 4.72, *P* < .001, $\eta_{p}^{2}$ ≥ 0.48, −31%, respectively; Figure 3).

## 5 DISCUSSION

This is the first study to compare the mechanical output from eccentric contractions of hamstring muscles as measured with IKD or NHD, while controlling relevant biomechanical parameters. In agreement with a previous observation,^10^ IKD and NHD testing yielded substantial differences. Intriguingly, the systematic bias of peak torque differences was found opposite to that previously reported (Figure 2A compared to the results based on Figure 4 in van Dyk et al^10^), when biomechanical parameters were uniformly controlled. In addition, torque differences were more pronounced for stronger individuals and we observed a considerable random error with a very weak relationship between IKD and NHD measurements (*r* < 0.58). This corroborates that current diagnostic devices, even if very similar in test design, reflect different determinants of hamstring muscle strength and methodological differences contribute to the apparent incongruity between various investigations (see Al Attar et al^2^ for review). Furthermore, current diagnostic devices of hamstring strength testing revealed a limited sensitivity to detect small yet meaningful strength differences within individuals (MDC_95_ > 15%), while values for angle of peak torque and strength symmetry are not acceptably reproducible.

### 5.1 Concurrent validity

Despite the adjustments made to match testing conditions between IKD and NHD tests, considerable differences were observed between the two methods, with lower eccentric torque in the isokinetic exercise compared to the Nordic hamstring exercise testing. These findings challenge our hypothesis, although the underlying mechanisms remain partly elusive.

The conflicting finding compared to the results in van Dyk et al^10^ and the low eccentric torque (~110 Nm) on the IKD compared to similar observations from individuals exposed to exercises on an isokinetic dynamometer (~170‐180 Nm),^10^, ^16^ is presumably predominantly elucidated by the hip‐dependent effects on the length‐tension relationship of the hamstring muscle. Measures in a position of hip flexion — a position that is still common in isokinetic measurement^10^, ^22^, ^30^ — cause greater hamstring muscle length during IKD movements and can increase the obtained eccentric hamstring torque by a factor of ~1.5.^31^ We have not conducted functional tests that might help to predict some transfer effects to athletes’ performance, but knowing that competitions in various sports or daily activities hardly require deep hip flexion, it seems rational that a measure with an extended hip has been considered the most appropriate method in relation to the physiological muscle length‐tension relationship.^32^ Moreover, isokinetic measures at extended hip positions most closely imitate the Nordic hamstring exercise, a key circumstance for the test mode comparison.

However, according to this standardized approach, the detected inter‐device torque difference contradicts the bilateral force deficit^33^ and could not be explained by the force‐length relationship,^34^ movement velocity, or different muscle activation (Figure 3). Presumably, due to individual daily functional requirements,^18^ the angle of peak torque varied widely between subjects, but interestingly, angles were systematically lower in NHD than IKD testing (Figure 2C). Yet, interpretations should be made with caution, because of the difficulty to assess angle parameters’ reliability using current diagnostic tests (Table 1). However, this finding remains appealing as it suggests that NHD tests contain the risk to not determine the eccentric peak torque. Thus, in situ studies indicated that muscle groups generally tend to produce maximum torque at a specific joint angle but show significant torque decrements outside this range.^35^ If this holds true for eccentric muscle contraction of Nordic hamstring exercise, the gravity‐induced low angle of peak torque of NHD measures could cause substantial underestimation of individual strength capacity. This uncertainty may contribute to the high random error and the very poor within‐subject correlation (*r* < 0.58) between the current diagnostic devices, but fails to explain the high proportional biased torque of NHD compared to IKD measures (Figure 2A). Speculation concerning the latter remains beyond the scope of this study, but the findings suggest that biomechanical principles influence the results differently. The similar results of NHD_30_ and NHD_max_ tests indicate a negligible bias of distinct, but low, Nordic hamstring exercise forward lean velocities (~5‐35° s^−1^). In contrast, the comparison of IKD and NHD measures (Figure 2A‐C) and their considerable typical error of the estimates (Figure 2D‐F) have shown for the first time that device‐specific differences beyond the hip position and movement velocity are sufficient for current diagnostic devices to measure different traits. These differences are also reflected in different patterns of muscle activation with lower gastrocnemii activity in NHD measurements. The effect of these activation differences cannot be estimated, but is consistent with the region‐specific muscle activation patterns during common hamstring exercise,^36^ and obviously, this neurological component can significantly affect knee joint torque development. In summary, these findings have important clinical and practical significance.

Practitioners and sports injury researchers should be aware of the likelihood that there is not a single training approach for hamstring prophylaxis or rehabilitation^37^ and that hamstring muscles appear to be too complex in nature to be amenable to a single diagnostic assessment. Ignoring this circumstance can have serious consequences on the expected outcome of experimental and correlational/cross‐sectional research. Accordingly, there is a danger that appraisals of the clinical or practical relevance of an exercise may be to a higher extent determined by the similarity of the exercise to the methodological diagnosis rather than actually contributing to improving hamstring protection or performance of an athlete. Therefore, the danger is that the evaluation of the clinical as well as the practical relevance of exercise is based mostly on similarities of exercises and methodological diagnostics, rather than actually contributing to the protection of the hamstring or the improvement in athlete's performance.

Concisely, in the worst case, the effect of the same exercise could be considered either insignificant or even be included in exercise guidelines for hamstring protection or athletic performance depending on the methodology and availability of the diagnostic device. While this is yet to be determined, recent reports of dissimilar sensitivity and specificity of the IKD and NHD to detect the risk of future hamstring strain injury (for review^2^, ^38^) should always be considered in relation to the included cohorts (eg, soccer, football, rugby, and sprinting) and the discrepancy of the used methodology between research groups.

### 5.2 Reliability and assessing individuals

No learning effect was found for eccentric peak torque measurements, and in fact, the ability to detect group differences or changes in hamstring strength is given when essential precautions are taken (Table). However, strength imbalance ratios and angle of peak torque measures revealed a very poor relative and absolute reliability on either diagnostic device. Similarly, the total eccentric work should not be evaluated using an NHD. Caution is also required when comparing individuals or individual adjustments. Accordingly, the high intra‐device MDC_95_ values (~15%‐20%) indicate that greater changes in knee flexor strength are necessary to detect real changes within individuals. For example, in the application of a critical force value of 337 N as an indicator of the future risk of football players,^12^ individual changes lower than approximately ±50 N could also be within the measurement error. When this 337 N force level is calibrated (assumed shank length of 0.4 m) and transformed to the criterion measure of isokinetic torque, the critical torque value and the boundaries of assessment error would be about 111 ± 17 Nm. Hence, both devices may be acceptable to detect the large response to loading usually observed after rehabilitation phases, but seem inappropriate to examine individual effects of preventive exercise in healthy athletes and must be considered with caution with regard to critical values of a risk of future injury.

### 5.3 Limitations

It should be noted that the IKD is a surrogate for true hamstring strength and represents a less‐than‐perfect instrument to use as a criterion.^30^ This was not corrected as the contribution to variability likely correlates with the IKD measurement. Hence, it was not our aim to convert a strength assessment of one device to the other, but show the error between them. If the regression coefficients (Figure 2A) are used, a conversion of NHD measures to IKD results seems only appropriate given a similar population and a similar IKD setting.

Moreover, our measurements of muscle activation during IKD and NHD were only based on local estimations from a single pair of EMG electrodes. The neuronal component of different muscle activations of the knee flexor muscles may have been better reflected by high‐density surface electromyography measurements, performed over whole muscle groups. Future studies should include such measurements.

## 6 CONCLUSION

The present study indicates that IKD testing and NHD testing bring about divergent estimations of eccentric hamstring strength and both methods do not reflect hamstring eccentric contraction in the same way. In terms of practical use, a familiarization appointment seems sufficient, and the influence of the kinematic control of the knee angular velocity during NHD testing appears to be negligible if the forward lean velocity remains slow (~5‐35° s^−1^). In contrast, the hip angle position has a profound effect on the measurement of eccentric knee flexor strength^31^ and should be strictly standardized and controlled in isokinetic and Nordic hamstring strength testing. Concerning individual diagnoses, the high MDC_95_ value (≥15%) suggests that training, prevention and/or rehabilitation recommendations often fall within a random variation in between‐session performance. Hence, only large intra‐subject differences of eccentric hamstring strength are detectable on either device. Furthermore, current diagnostic devices are not suitable to reliably determine the angle of peak torque and bilateral eccentric knee flexor strength imbalance.

## 7 PERSPECTIVES

Methodological heterogeneity limits our understanding, and a larger consensus on methodologies used to test knee flexor strength is required. Another gap revealed by the present study relates to the probability that NHD measures are performed in a range of motion that does not include the actual angle of peak torque. The NHD should not be deprived of its strength in practical use, but it appears necessary to consider assistant systems^2^ that enable a rating of a larger range of motion. This would potentially increase the validity of NHD measures and reduce the risk of bias in recommendations for injury prevention or rehabilitation monitoring. Nonetheless, future research endeavors should also consider implementing multifactorial strength assessment to increase the sensitivity of measurement and therefore improve its prevention and prediction methods. Future studies should assess the influence of these limitations on the assessment of training adaptation.
